# Chloride promotes refolding of active *Vibrio* alkaline phosphatase through an inactive dimeric intermediate with an altered interface

**DOI:** 10.1002/2211-5463.12565

**Published:** 2018-12-21

**Authors:** Jens Guðmundur Hjörleifsson, Bjarni Ásgeirsson

**Affiliations:** ^1^ Department of Biochemistry, Science Institute University of Iceland Reykjavik Iceland

**Keywords:** alkaline phosphatase, bimane fluorometry, dimer, half of sites, urea denaturation, *Vibrio splendidus*

## Abstract

Most enzymes are homodimers or higher order multimers. Cold‐active alkaline phosphatase from *Vibrio splendidus* (VAP) transitions into a dimer with very low activity under mild denaturation conditions. The desire to understand why this dimer fails to efficiently catalyse phosphomonoester hydrolysis led us to investigate interfacial communication between subunits. Here, we studied in detail the unfolding mechanism at two pH values and in the presence or absence of sodium chloride. At pH 8.0, the denaturation model had to include an inactive dimer intermediate and follow the pathway: *N*_2_ → *I*_2_ → 2*U*. At pH 10.5, the model was of a two‐state nature. Enzyme activity was not recovered under several examined refolding conditions. However, in the presence of 0.5 m NaCl, the enzyme was nearly fully reactivated after urea treatment. Thermal inactivation experiments were biphasic where the inactivation could be detected using CD spectroscopy at 190–200 nm. By incorporating a bimane fluorescence probe at the dimer interface, we could monitor inactivation/denaturation at two distinct sites at the dimer interface. A change in bimane fluorescence at both sites was observed during inactivation, but prior to the global unfolding event. Furthermore, the rate of change in bimane fluorescence correlated with inactivation rates at 40 °C. These results indicate and support the hypothesis that the subunits of VAP are only functional in the dimeric state due to the cooperative nature of the reaction mechanism when proper crosstalk between subunits is facilitated.

**Abbreviations**
AP: alkaline phosphatase
CD: circular dichroism
ECAP: *E. coli* alkaline phosphatase
GdmCl: guanidinium chloride
mBrB: mono‐bromo bimane
PLAP: placental alkaline phosphatase
pNPP: para‐nitrophenyl phosphate
SEC: size exclusion chromatography
TrIQ/TyrIQ: tryptophan‐ and tyrosine‐induced quenching
VAP: *Vibrio* alkaline phosphatase

Quaternary protein structures provide additional ways to modulate the dynamical ensemble of conformations that accompany the reaction pathway in multimeric proteins. Finding how enzyme subunit interactions affect function in each case is an experimentally challenging task. The benefit of homodimer formation has been attributed to several factors, including increased stability by formation of interface contacts 1, 2, extra sites becoming available for ligand binding 3, faster folding being facilitated that minimizes occurrence of misfolded intermediates 4 and the provision of mechanisms for allosteric regulation 5. Many homodimeric enzymes show evidence of cooperativity 6, 7, 8, 9, 10, 11 and especially negative cooperativity resulting in half‐of‐sites reactivity 8, 10. Thus, the role of having two identical subunits is in many cases to increase the conformational space through a dynamical asymmetry, coordinated by interface contacts 12, 13, 14.

Several earlier experiments were conducted in order to answer the question of ‘subunit individuality’ in alkaline phosphatases [APs, (EC3.1.3.1)]. Bloch and Schlesinger (1974) hybridized an inactive *Escherichia coli* AP (ECAP) variant with the wild‐type, which showed half the activity of the wild‐type enzyme 15. This suggested that only one subunit was needed for catalytic activity. However, inactive monomers have been frequently observed as a part of ECAP dimers, indicating that monomers are not independent 16, 17, 18, and the monomers by themselves are generally inactive. Thus, these results do suggest that an interface is needed to properly form the active sites by some global residue‐positioning induced through the interface.

We have previously shown that an AP from the marine bacterium *Vibrio splendidus* (VAP) can adopt different conformers by bringing the pH from 8.0 to 10.5 and that VAP's activity and stability are greatly affected by salt ions 19. Furthermore, we have discovered that VAP is particularly sensitive to heat (room temperature) in dilute buffers or low concentration of urea. Furthermore, the form of the urea‐inactivated enzyme (at 1.0 m urea) seems to be fully metallated with two Zn^2+^ and one Mg^2+^ per monomer and still exists as a dimer 19, 20. This has brought us to investigate further the nature of the inactivated dimer, which could shed light on how the subunits might interact asymmetrically during catalysis. In fact, under equilibrium conditions, APs have been shown to populate several intermediate forms during both unfolding and refolding 21. For ECAP, the active site of the enzyme can be reactivated using different pathways/protocols 22. However, upon denaturation of monomers, most APs (ECAP is the exception) do not recover the activity or fail to dimerize 23.

We decided to investigate in detail the denaturation and refolding of VAP at various conditions, in order to detect intermediate states and determine quaternary structure under each condition. Throughout, we performed measurements both at pH 8.0 and at pH 10.5, since we have shown that the enzyme occupies distinct states at these pH values due to a deprotonation event of an unknown group 19. To focus on changes at the dimer interface upon inactivation/denaturation, we introduced a fluorescence probe, bimane, at the interface. Bimane is intrinsically quenched by Trp and Tyr within a defined sphere of 15 Å for Trp 24 and 10 Å for Tyr 25. Optimally, a phenylalanine residue should act as a nonquencher control for the Trp/Tyr quencher positions 25. Thus, we rationally incorporated bimane at the dimer interface juxtaposing either Tyr or Trp on the other monomer to probe for subtle changes at the dimer interface during inactivation. Results indicated that upon inactivation by urea or heat the enzyme is transitioned to an inactive dimer where interface interactions were altered.

## Results

### Urea denaturation of VAP, effect of NaCl and pH

**Figure 1:**
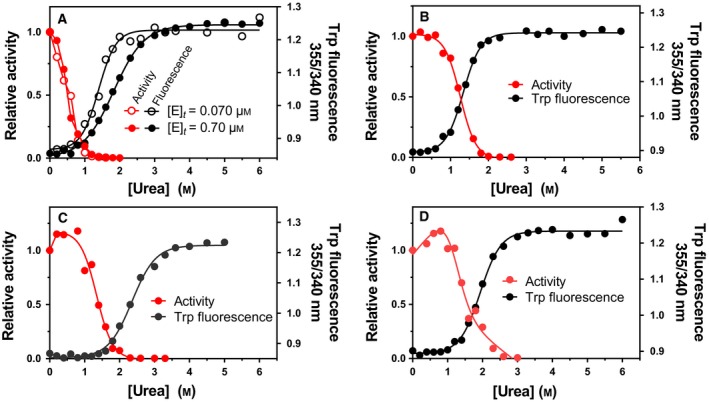
Urea denaturation of VAP as monitored by Trp fluorescence and residual activity. Urea denaturation was performed at pH 8.0 (A, C) and pH 10.5 (B, D) with 0.5 m NaCl (C, D) or without NaCl added (A, C). At pH 8.0, the effect of enzyme concentration was tested at [*E*]_*t* _= 0.70 μm and 0.070 μm (A).

Measuring VAP denaturation by following Trp fluorescence as a function of urea showed that denaturation of the folded state follows a two‐state mechanism (Fig. 1A). The loss of activity was a prior event to the unfolding transition monitored by intrinsic fluorescence. Thus, the minimal denaturation model was a three‐state model including an inactive intermediate state of the dimer. Urea unfolding was performed at different enzyme concentration to address which transitions were protein concentration dependent (Fig. 1A). The Trp fluorescence curve showed concentration dependence, thus reflecting a transitional equilibrium of a folded dimer towards two unfolded subunits. The simplest denaturation model at pH 8.0 was therefore found to be: *N*_2_ → *I*_2_ → 2*U*,* N*_2_ being the native state, *I*_2_ the dimer intermediate and U unfolded monomers. The effect of NaCl (Fig. 1C,D) and of raising the pH to 10.5 (Fig. 1B,D) was tested. At pH 8.0, the Δ*G* for the inactivation step (*N*_2_ → *I*_2_) was only 2.7 kJ·mol^−1^ (less than the strength of one hydrogen bond) but was stabilized to 9.8 kJ·mol^−1^ with 0.5 m NaCl present (Fig. 5B and Table 1). At pH 8.0, the Δ*G* for the *I*_2_ → 2*U* unfolding transition was 48 kJ·mol^−1^ and 65 kJ·mol^−1^ without or with 0.5 m NaCl present, respectively. These values for the dimer dissociation are in agreement with the values derived by monitoring the specific activity as a function of enzyme concentration in our previous study 19.

**Table 1:** Standard free energy for the denaturation of VAP by urea. Activity vs. urea was used to probe the formation of the inactive dimer intermediate at pH 8.0. Trp fluorescence vs. urea was used to monitor the dissociation/unfolding of the dimer. At pH 10.5, the activity overlapped with Trp fluorescence transitions as a function of urea and could thus also be used for monitoring the dissociation/unfolding of the dimer. At pH 8.0, the buffer composition was 20 mm Mops, 1 mm MgSO_4,_ and at pH 10.5, it was 20 mm Caps, 1 mm MgSO_4_. The midpoint of denaturation (D_1/2_) for each transition is shown in brackets. Data are presented as mean ± SD

	Conditions	Δ*G* (kJ·mol^−1^) *N*_2_ → *I*_2_	Δ*G* (kJ·mol^−1^) *I*_2_ → *N*_2_ (pH 8.0) *N*_2_ → 2*U* (pH 10.5)	Δ*G* (kJ·mol^−1^) *N*_2_ → 2*U* (activity)
pH 8.0	Buffer	2.7 ± 0.3 [0.5 m]	48 ± 2 [1.8 m]	–
	Buffer + 0.5 m NaCl	9.8 ± 1.6 [1.4 m]	65 ± 4 [2.5 m]	–
pH 10.5	Buffer	–	57 ± 1 [1.2 m]	58 ± 5 [1.2 m]
	Buffer + 0.5 m NaCl	–	62 ± 2 [1.6 m]	51 ± 6 [1.6 m]

At pH 10.5, the active‐site stability of VAP towards urea was enhanced due to a conformational change 19, with the result that the enzyme was inactivated and unfolded at the same time. Thus, the model for inactivation became a two‐state process (*N*_2_ → 2*U*). The inactivation and Trp fluorescence transition overlapped and either activity or Trp fluorescence transitions could reflect the total Δ*G*. The Δ*G* at pH 10.5 was estimated at 57 kJ·mol^−1^ as monitored by Trp fluorescence and 58 kJ·mol^−1^ using activity. With the addition of 0.5 m NaCl at pH 10.5, there was only slight change in this Δ*G* value reflecting an increase in the denaturation midpoint from 1.2 m to 1.6 m. Interestingly, there was a gradual increase in activity at urea concentration in the range 0–1.0 m with 0.5 m NaCl present which peaked at 120% of the initial activity. This was seen both at pH 8.0 and at pH 10.5 (Fig. 1C,D). Similar activation has been reported for other APs induced by low GdmCl concentrations 26, 27.

### Refolding and reactivation are dependent on pH and NaCl

**Figure 2:**
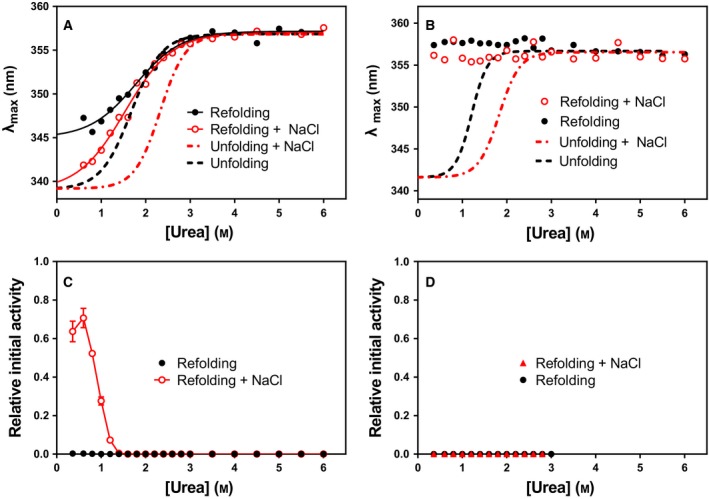
Refolding of VAP after denaturation in 6 m urea. Enzyme sample at 0.6 mg·mL^−1^ was incubated in 6.0 m urea for 16 h and then diluted by a factor of 15x to a buffer containing 0.35–6.0 m urea. Trp fluorescence was monitored (A) at pH 8.0 and (B) at pH 10.5 with and without 0.5 m NaCl. Unfolding traces from Fig. 1 are shown as dotted lines. Activity was monitored using the standard activity assay and normalized to activity of sample incubated without 6.0 m urea, for pH 8.0 (C) and pH 10.5 (D), with and without 0.5 m NaCl.

Reversibility of the unfolding of the enzyme was monitored by diluting it into lower urea concentrations in the range 0.35–6.0 m urea from 6.0 m urea (Fig. 2). At pH 8.0 (Fig. 2A), the unfolding was only partially reversible in the absence of NaCl but was nearly fully reversible when 0.5 m NaCl was added both to the 6.0 m urea buffer and to the refolding buffer. It was important to keep NaCl present in both buffers; otherwise, the recovery of activity was not as efficient upon refolding. However, the unfolding and refolding traces at 0.5 m NaCl did not overlap and had different midpoints of denaturation. This indicated that the enzymes adopted an intermediate state after unfolding with a kinetic barrier resulting in higher dilution of urea needed for proper folding. At pH 10.5, the denaturation was completely irreversible and NaCl had no effect on refolding efficiency (Fig. 2B). Interestingly, 70% of the activity of the enzyme was recovered at pH 8.0 with 0.5 m NaCl present, but no activity was detected without NaCl present, even though the Trp fluorescence measurements (judged by a blue shift in λ_max_) indicated partial folding towards the native state, no activity was recovered (Fig. 2C). Correspondingly, there was no recovery of activity at pH 10.5 (Fig. 2D).

**Figure 3:**
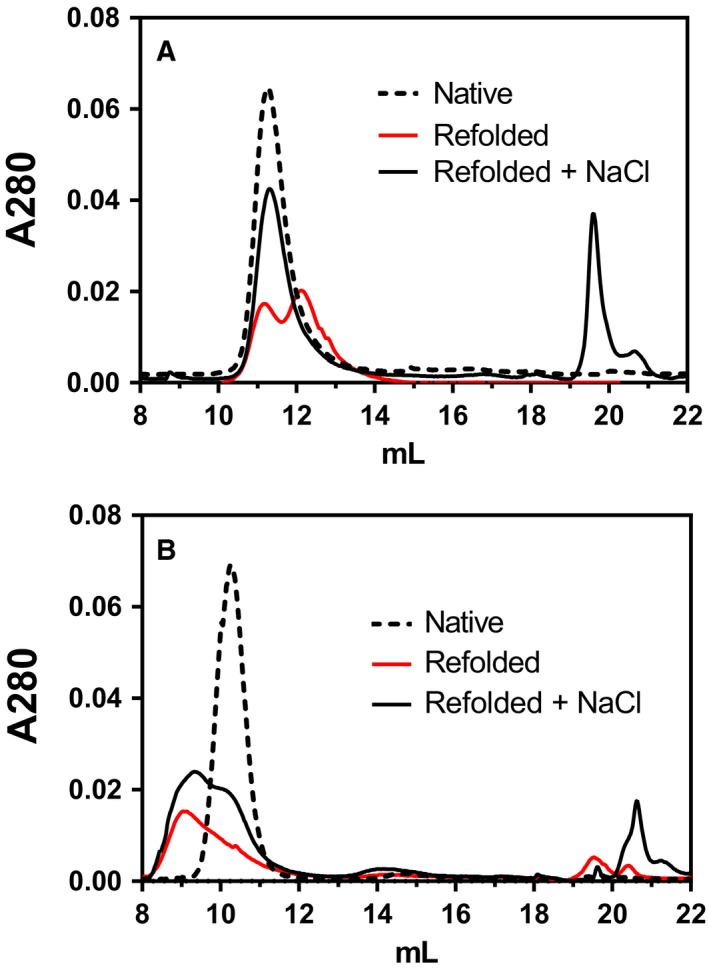
Analytical size exclusion chromatography on Superose 12 column after denaturation of VAP and attempted refolding. Concentrated VAP samples from a 24 mg·mL^−1^ stock were incubated for 16 h at 10 °C in 3.0 m urea before being diluted by a factor of 15x giving final concentration of 0.2 m urea, 0.5 mg·mL^−1^ enzyme. Then, diluted samples were further incubated for 4 h at 10 °C. The sample and running buffers were either (A) 20 mm Mops, 1 mm MgSO_4_, pH 8.0 or (B) 20 mm Caps, 1 mm MgSO_4_, pH 10.5. Native controls (dotted lines) were incubated for 4 h in the running buffer prior to injecting to the column.

We used analytical size exclusion chromatography (SEC) to determine the quaternary form of VAP after urea denaturation and subsequent dilution to facilitate refolding. Here, 3.0 m urea was used instead of 6.0 m urea, simply due to the high concentration of enzyme needed to reach 0.5 mg·mL^−1^ of enzyme after dilution to 0.2 m urea. This is, however, a sufficiently high concentration of urea to fully unfold the enzyme. At pH 8.0, after dilution of urea (Fig. 3A), only about half of the enzyme populated a dimeric state and the other half a monomeric state (red trace). Furthermore, this dimeric state was inactive and is likely the dimeric intermediate state (*I*_2_) observed in Fig. 1A,C. An intriguing result was seen with 0.5 m NaCl present at pH 8.0. About 65% of the enzyme molecules adopted dimeric state (~ at 11 mL in Fig. 3A) which correlate well with the 70% activity recovery seen in Fig. 2C, but the rest of the population eluted out of the Superose 12 column in the total volume fraction (*V*_t_) as two peaks (~ at 20–21 mL in Fig. 3). This observation was repeatable, and measurements were made from the same batch as for the native control, where no such peaks were observed in the *V*_t_ fraction. Furthermore, when only buffer was injected, no background from a possible contamination was observed. The *V*_t_ peaks could be small peptides from degradation, some preferably interacting with the column matrix since part of the peak was observed after *V*_t._ At pH 10.5, the enzyme adopted mostly an unfolded monomeric species with a broad peak around 8–12 mL. Again, as was observed at pH 8.0 with addition of NaCl, peaks near *V*_t_ were observed. At pH 10.5 in Caps buffer, the native dimer control eluted at 10.3 mL versus 11.3 mL at pH 8.0. Also, for the refolded samples at pH 10.5, a small population of an intermediate peak is observed at 14.0 mL.

### Multiphasic thermal inactivation of VAP

**Figure 4:**
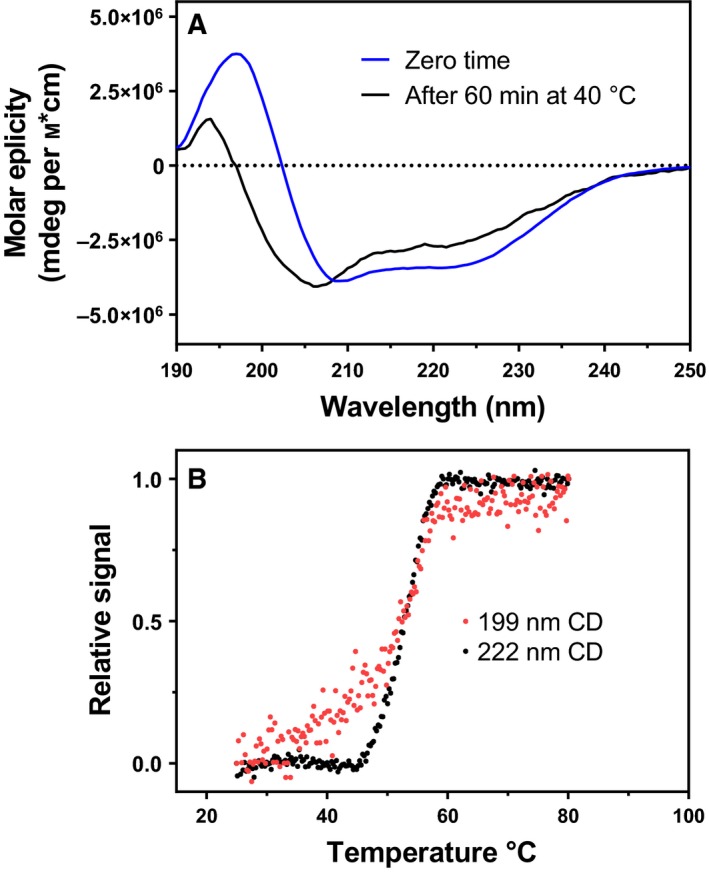
Thermal inactivation of VAP as monitored by CD spectroscopy. (A) CD spectra in 10 mm borate, 10 mm MgCl_2_, pH 8.0, was measured at 40 °C at zero time and after 1 h incubation at 40 °C. (B) VAP melting temperature monitored at 199 nm and 222 nm under same conditions as in (A), using a 1.0 °C·min^−1^ temperature gradient. The concentration of enzyme sample was 0.3 mg·mL^−1^, and the samples were measured in a 1‐mm cuvette. The signal was normalized to the difference between the signals at 70 °C and 25 °C.

Thermal inactivation of VAP is classically thought of as a two‐state process 28, 29, 30. By monitoring the thermal denaturation using circular dichroism spectroscopy (CD) at 222 nm, a two‐state melting temperature for the folded state was observed. We decided to check in detail the CD spectrum of VAP in the range 190–250 nm, both in the native state and after thermal inactivation at 40 °C (Fig. 4). Under the latter conditions, the enzyme is still mostly folded but inactive judged by CD at 222 nm and activity assay respectively, performed 1 h after incubation. Using our typical Mops or Tris buffers at pH 8.0, we were unable to get data below 205 nm due to absorption of the buffer molecules. A preferred buffer for use in CD measurements is phosphate, due to its low UV absorption. However, it is ill‐suited for APs since it is a strong competitive inhibitor and stabilizer. Borate (tetraborate) is a much weaker competitive inhibitor and can be used as a buffer at moderate concentration without interacting with the enzyme to a great extent 23. Using borate as a buffer ion, CD spectra could be measured at 190–250 nm. A relatively large change at 190–200 nm was observed upon inactivation at 40 °C (Fig. 4A), peaking at 199 nm. At 40 °C, the enzyme was nearly inactive (after 1 h incubation) but only a small fraction of the enzyme was unfolded. This observation indicated a conformational change during inactivation. By monitoring both 199 nm and 222 nm at the same time, two melting transitions could be observed (Fig. 4B). The former transition showed less cooperative character (steepness) than the second unfolding transition, but merged with the unfolding event at 45–60 °C.

**Figure 5:**
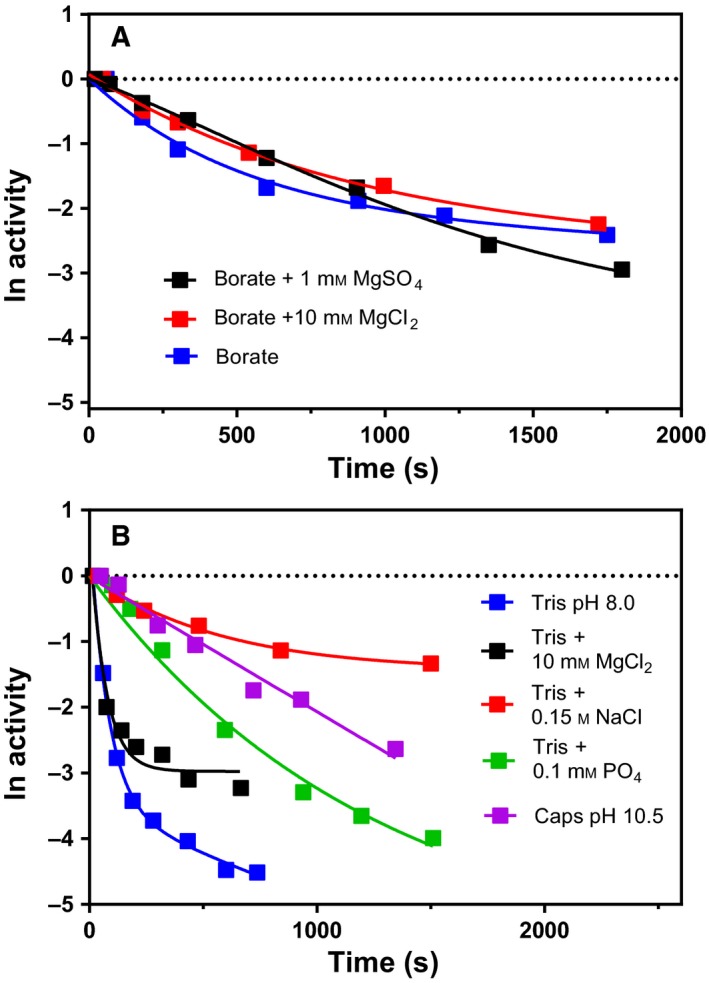
Thermal inactivation of VAP under different buffer conditions. Enzyme samples were incubated at 40 °C and the activity measured using the standard activity assay. (A) Effect of MgSO_4_ and MgCl_2_ in 10 mm borate buffer pH 8.0. (B) Effect of MgCl_2_, ${\text{HPO}}_{4}^{2-}$, NaCl and pH (8.0 vs. 10.5). With the natural logarithm of activity numbers on the y‐axis, the data were fitted with a single exponential function except for the samples at pH 10.5 in Caps buffer which was fitted using linear regression (1 exponent).

When we tested the thermal stability of the active site in 10 mm borate by measuring substrate hydrolysis, we found to our surprise that the inactivation seemed to be a mixture of two events (double exponential in Fig. 5A). When looking further into the inactivation of VAP under the previously used assay conditions (10 mm Tris, 10 mm MgCl_2_, pH 8.0), we found that if the inactivation was followed down to 5–10% residual activity, a clear curvature in the ln(activity) vs. time was observed (Fig. 5B). Previously this had been overlooked since the activity was rarely assayed under 10% of initial activity under the typical assay conditions of 20 mm Tris, 10 mm MgCl_2_, pH 8.0. The effect of MgCl_2_, NaCl and competitive inhibitor ions (${\text{SO}}_{4}^{2-}$ and ${\text{HPO}}_{4}^{2-}$) on the inactivation rate constants is shown in Table S1. NaCl, MgSO_4_ and ${\text{HPO}}_{4}^{2-}$ decreased the rate of *k*_1_ but only NaCl decreased the rate of inactivation for both *k*_1_ and *k*_2_. Increasing the pH to 10.5, the curve becomes single exponential with no active intermediates, similar to the results seen for urea denaturation at pH 10.5 (Fig. 1B,D).

### Probing structural changes at the dimer interface during inactivation

*Vibrio* alkaline phosphatase was labelled with the fluorescence probe bimane in two positions at the dimer interface, where either Trp or Tyr could serve as intermolecular quenchers in a distance‐dependent manner 24, 25. The dimers of APs have a twofold symmetry with an isologous interface. Thus, residues at the interface are not in symmetric positions except close to the twofold rotational axis. The Cys residues that we introduced in each monomer and used to attach the bimane probe did not face each other and form a disulphide.

**Figure 6:**
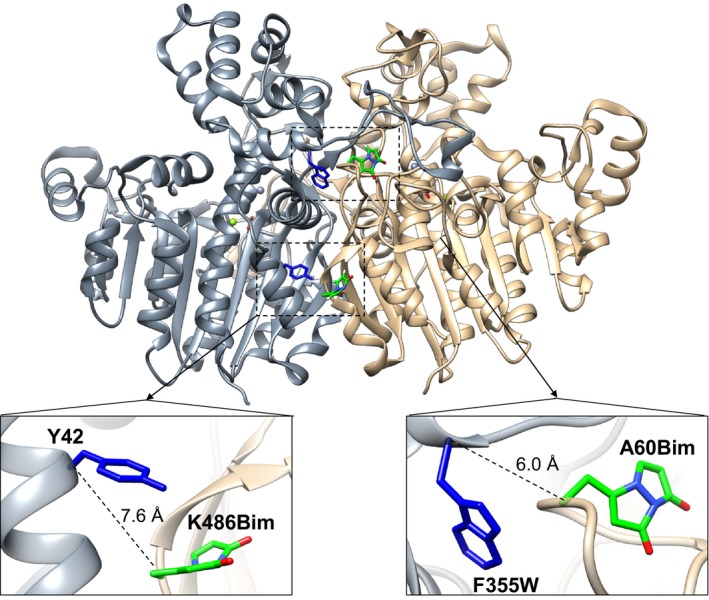
Incorporation of the bimane fluorophore at the dimer interface. Cys mutations were made to label VAP selectively with mono‐bromo bimane. Bimane was attached at position K486C (lower left inset) and A60C (lower right inset). The label at K486Bim juxtaposed Y42 at the other subunit with α‐α carbon distance of 7.6 Å. At A60Bim Trp was incorporated at position F355 (F355W) at the other subunit with Cα‐Cα distance of 6.0 Å. At these distances, Tyr and Trp should act as intermolecular fluorescence quenchers of bimane. The bimane molecule was built into the VAP crystal structure (PDB ID: 3E2D) using the *build structure* tool in UCSF Chimera without spatially altering native rotamers.

The first labelling site that was chosen is located on a short loop juxtaposing F355 at the base of the large interface loop of the other monomer (Fig. 6, right inset). This site was chosen due to the fact that the enzyme variant F355W has been found to have identical kinetic properties as the wild‐type VAP 20 and the native residue could act as a nonquenching control. Cys was introduced at position A60 with a Cα‐Cα distance to F355 in the other monomer of 6.0 Å. Phe355 was also replaced by a Trp355 (A60C/F355W) to act as an intrinsic quencher.

The second site that we studied was at K486 where a Cys was incorporated. This residue has a Cα‐Cα distance of 7.6 Å to Y42 of the other monomer (Fig. 6, left inset). The Y42F variant was used as a control for the tyrosine‐induced quenching (K486C/Y42F), which additionally would result in loss of one hydrogen bond between K486 and Y42.

*Vibrio* alkaline phosphatase has one native Cys residue in location 67 which did not react with the label in the native state due to low accessibility. This was convenient, since the C67S variant that would have been needed in order to introduce bimane singularly at other sites has 40% lower *k*_cat_ than the wild‐type VAP 31. However, with the enzyme unfolded in 4.0 m urea, the C67 site could be nearly 100% labelled.

We have previously observed that the exchange of Cys for residues at various sites in VAP decreased the activity of the enzyme substantially 31. Here, all of the Cys replacements decreased the *k*_cat_ and most of the substitution increased the *K*_M_ (Table S2). The K486C substitution had the least impact on *k*_cat_ compared to the other interface variants, being 65% active compared with the wild‐type and displaying a slightly reduced *K*_M_. Furthermore, when the bimane label was introduced at the K486C site, both the *k*_cat_ and the *K*_M_ increased. This variant was similar to the wild‐type without the bimane attached as regards to the space occupied by the side‐chain group. In the double variant K486C/Y42F, the *k*_cat_ decreased further compared to K486C but the *K*_M_ remained similar. The Y42F mutation by itself did not affect the enzyme kinetics. A more dramatic decrease in activity was seen for A60C/F355W, where *k*_cat_ was only 2% of the wild‐type and the *K*_M_ increased by 60% resulting in a *k*_cat_/*K*_M_ of less than 1% compared to the wild‐type. Interestingly, the F355W mutation on its own had no effect on the kinetic constants but lost its neutrality when accompanied by the second mutation in A60C/F355W. This pairing had a dramatic effect on the functional parameters for catalysis (see Table S2).

Incorporation of a Trp residue to position 355 (F355W/A60Bim) had a small effect on bimane emission decay, where the average lifetime <τ> (composed of the sum of three lifetimes and their amplitudes) decreased from 6.9 ns to 6.6 ns, indicating that the dynamic quenching component of Trp (F355W) was small. However, when measuring the emission decay of the unfolded A60Bim/F355W variant, the average lifetime increased to 10.0 ns (Table S3), indicating that in the folded state the bimane probe attached to A60C was likely quenched by other means (main chain or other residues). Furthermore, the steady‐state emission spectra of A60Bim compared to A60Bim/F355W showed that the emission increased slightly when F355W was introduced (Fig. S1). This increase in emission might be caused by the bimane probe being more buried and excluded from the solvent in the A60Bim/F355W variant, since the λ_max_ decreased from 468 nm to 464 nm. A similar trend was seen for the K486C bimane‐labelled variant. Comparing the steady‐state emission of K486Bim (with Y42 as quencher) and K486Bim/Y42F (absence of quencher), there was little change in emission or in the emission decay (Fig. S1).

**Figure 7:**
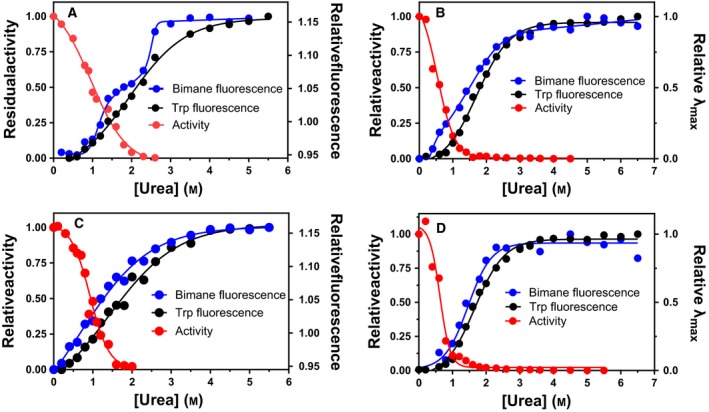
Urea denaturation of bimane‐labelled VAP variants. Bimane fluorescence at interface was compared to Trp fluorescence and activity for variants (A) A60Bim/F355W, (B) A60Bim, (C) K486Bim and K486Bim/Y42F (D). The two top figures (A, B) have an intrinsic quencher of bimane present (Trp or Tyr) in proximity of the bimane label, while the bottom two graphs (C, D) the quencher is absent. Transitions were monitored by the change in the intensity ratio of 355/340 nm and 468/475 nm for Trp and bimane fluorescence, respectively (right axis), in (A, C) but by the relative shift in λ_max_ for K486Bim and K486Bim/Y42F (B, D).

Despite the fact that both the fluorescence intensity and lifetime did not change when the intermolecular intrinsic quencher (Tyr or Trp) was introduced inside bimane sphere of quenching 25, we were interested to see whether we could detect structural changes by monitoring bimane fluorescence induced by urea denaturation. Since the bimane probe is very sensitive to changes in distances of the probe relative to quencher (Tyr/Trp), a change in distance as short as 1.0 Å should in theory be detectable. Furthermore, the solvent accessibility (indicated by the λ_max_ of fluorescence) can also be used to probe structural changes. As shown above, the urea denaturation model for VAP at pH 8.0 is *N*_2_ → *I*_2_ → 2*U*, where a dimeric intermediate with greatly impaired activity is populated. It was not possible to monitor the first transition by total Trp fluorescence. However, by following the bimane fluorescence as a function of urea, a three‐state curve was obtained where *I*_2_ could be detected (Fig. 7). This could either be observed by monitoring the absolute fluorescence for A60Bim/F355W (Fig. 7A) or by following the λ_max_ for the K486Bim variant (Fig. 7B). Interestingly, without the Tyr or Trp quencher juxtaposing the bimane label (Fig. 7C,D), the transition was not detected, indicating that the observed changes in bimane fluorescence were due to adjustments in the orientation of the quencher/probe pairs.

**Figure 8:**
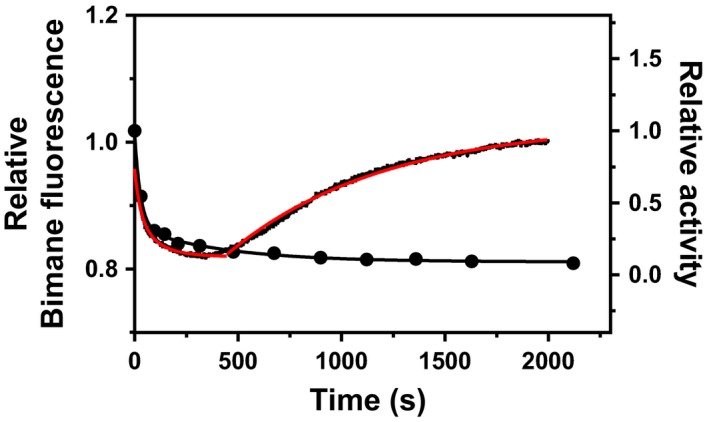
Inactivation monitored by fluorescence of the K486Bim variant. Relative fluorescence (left *y*‐axis, red trace) represents the relative maximal fluorescence at 435 nm using 380 nm as excitation. Activity was monitored under identical conditions (right‐axis close circles) where the numbers represent activity relative to activity at *t *= 0.

We also monitored bimane fluorescence during thermal inactivation at 40 °C (Fig. 8). By monitoring the K486Bim variant, two transitions could be observed with rate constants of 2.1 min^−1^ and 0.078 min^−1^ which correlated well with the inactivation rate constants previously observed in 20 mm Tris, 10 mm MgCl_2_, pH 8.0 (Table S1), that were 2.0 min^−1^ and 0.086 min^−1^, respectively. The first transition resulted in a decrease in fluorescence, while the second contributed to an increase in fluorescence. Thus, the first transition needed to be fitted to second‐order exponential decay, while the second transition could be fitted with a single exponent from 500 to 2000 s.

## Discussion

### VAP reversibly refolds in the presence of NaCl

We succeeded in showing for the first time that fully denatured VAP can be refolded back to the active form if maintained in the presence of 0.5 m NaCl. NaCl could be envisioned to work by stabilizing an intermediate structure in the folding process, by enriching a subtle state of the native structure with activity or by binding to the non‐native structure to prevent misfolding. Without NaCl present in the refolding buffer, but absent from the denaturant, the enzyme was shown to regain the tertiary structure, but the dimer failed to regain activity. In fact, it could be shown to be a mixture of monomers and dimers. This inactive dimeric state may be the last populated intermediate state towards full activation of the enzyme and NaCl decreasing its population on the timescale of folding. It has been generally accepted that bacterial APs fold and assemble their native dimers extracellularly in the periplasmic space. This necessitates the transport of the unfolded subunits over the plasma membrane by the SEC translocon 32, 33, 34. For marine organisms, the salt concentration in their oceanic habitat is expected to be in the 0.5–0.6 m range, and there is no evidence that NaCl concentration in the periplasm differs from the extracellular space. Thus, VAP has possibly evolved to utilize the environmental NaCl in its folding mechanism. The effect of NaCl on refolding of other APs needs to be evaluated to address if this only applies for APs from organisms living in high salt environments.

The effect of bringing the pH to 10.5 was shown to stabilize the enzyme towards transition from a fully active dimer to inactive dimeric state, but to our surprise the unfolding at pH 10.5 was not reversible upon removal of urea. We have previously proposed that at pH 8.0, a chloride ion from the addition of NaCl binds to a Zn ion in the active site and this might help maintaining active‐site integrity 19. Chloride binding to Zn is supported by results from Gettins and Coleman (1984) who observed a Cl^−^ coordinated to Zn ion at the M1 site in ECAP using ^35^Cl and ^113^Cd nuclear magnetic resonance (NMR) 35. Moreover, a chloride ion was observed bound to the Zn ion at site M1 in the crystal structure of an AP from the extreme halophile *Halomonas sp. 593*, which is structurally highly homologous to VAP. This is the same site where a phosphate ion (or sulphate ion) is normally coordinated in various AP crystal structures 36. Unlike the conditions used for the crystallization of VAP, no ${\text{SO}}_{4}^{2-}$ was used in the crystallization conditions of the halophilic AP. Thus, ${\text{SO}}_{4}^{2-}$ might have displaced a Cl^−^ during crystallization of VAP. A closely related AP from *Vibrio alginolyticus* was similarly activated by NaCl as VAP, and ${\text{SO}}_{4}^{2-}$ was shown to prevent the activation effect of Cl^−^. This indicated that Cl^−^ competes with ${\text{SO}}_{4}^{2-}$ at the same site 37. Taken together, the effect of NaCl on VAP activity is likely reflected in Cl^−^ binding to Zn in site M1. The mechanism of rate enhancement would be encapsulated in the faster displacement of the inorganic phosphate ion at the end of the reaction cycle. This would explain why chloride does not increase the activity of VAP at pH 10.5, since at pH 10.5 the affinity of the enzyme for the product has decreased to the point that the product release becomes faster than the hydrolysis step, which is unaffected by chloride. It could be insightful to see whether this kind of reaction mechanism involving chloride is general for all APs or only applies to some. We are currently optimizing crystallization conditions applying cocrystallization or soaking with NaCl, and devoid of competitive inhibitor ions such as sulphate and phosphate, to test this hypothesis.

### Biphasic thermal inactivation

Most enzymes are inactivated by heat in one transition, yielding a fully unfolded state. But in some cases, enzymes are first converted to an inactive but folded intermediate. This is in many cases observed for cold‐adapted enzymes, which tend to lose activity at lower temperatures than needed for unfolding of the polypeptide chain 38, 39, 40, 41. VAP is a clear example of a cold‐adapted enzyme losing activity at both low temperature and low urea concentration. Furthermore, the inactivation was shown here to be of a biphasic character involving two inactivation events occurring at the same time. The inactivation rate was dependent on competitive inhibitor ion concentration (phosphate, sulphate, borate), pH and NaCl concentration. Biphasic inactivation has been observed for heat inactivation of ECAP 42 as well as during GdmCl or urea inactivation of Atlantic cod AP or calf intestinal AP 26, 43. For the ECAP case, the two decay components had 50% of the amplitude each (activity) which indicated that the subunits of ECAP were asymmetrical in regard to stability. Another possibility might be that the inactivation was cooperative such that when one subunit is inactivated, a conformational change increases the stability of the other subunit. For VAP, the relative amplitudes for the fast (*k*_1_) and slow (*k*_2_) components were not identical. Furthermore, only the fast component was affected by competitive inhibitor ions. The fast component is likely the transition towards a mostly inactive dimeric intermediate state that is subsequently fully inactivated by a slower dimer dissociation (the slow component).

The CD spectra below 200 nm were very informative, as they changed greatly during VAP inactivation. In the far‐UV range (190–200 nm), absorption is mainly due to π → π* transitions of carbonyl groups. Thus, the changes observed would indicate loss of handedness of carbonyl groups, possible in ß‐sheets. Interestingly, this transition did not affect the hydrophobic packing, since the there was no change in solvent accessibility as judged by λ_max_ for Trp fluorescence. Nonetheless, it was clear that a structural change was being observed during inactivation by monitoring simultaneously CD at 199 nm and 222 nm during a temperature ramp of 1 °C·min^−1^ (Fig. 4B). It should be noted that the CD spectra were recorded in borate buffer, which is a weak competitive inhibitor previously shown to stabilize VAP (Fig. 5A). This does not, however, change the observed results that a local structural change was observed during VAP inactivation by heating.

### Dimer intermediate formation monitored by fluorescent probe at interface

The distance‐dependent quenching of bimane fluorescence by tryptophan or tyrosine (TrIQ/TyrIQ) was successfully used earlier to monitor hinge‐bending in lysozyme 25 and lid movements in *Thermomyces lanuginosus* lipase 44. By labelling lysozyme at different sites, it was well established that Trp and Tyr residues in proteins quench bimane fluorescence within a well‐defined spherical quenching radius 24, 25.

Bimane labels at sites A60C (A60Bim) or K486C (K486Bim) in VAP gave a local signal as the enzyme was inactivated (*N*_2_ → *I*_2_), but only when positioned inside the sphere of quenching of a Trp or Tyr located in the other monomer. Furthermore, both the fast (*k*_1_) and the slow (*k*_2_) components of inactivation were detected by changes in bimane fluorescence of the K486Bim variant, which correlated with inactivation rates. The fast component (*N*_2_ → *I*_2_) resulted in a decrease in fluorescence which indicated that the Y42 quencher of the other monomer might be moving closer. In that event, the quenching would be relieved by the slow component when the dimer dissociates. We can, however, not rule out the possibility that other intrinsic quenching effect(s) might be occurring, such as quenching by the backbone of the protein. However, it is clear that the observed effect was local at the dimer interface since no changes in Trp fluorescence could be detected in the same experiments.

### Interface ‘crosstalk’ and half‐of‐sites reactivity

The half‐of‐sites reactivity of APs, where one active site ejects the product as the other binds a new substrate through a reciprocal structural change, has been difficult to measure directly. A plausible explanation for the difficulty is that the half‐of‐sites structural oscillation must involve a subtle conformational change that is difficult to detect in a mixed state. During purification, APs usually have a phosphate occupied bound in both active sites, which needs to be removed by denaturation and dialysis before studies on occupancy in relation to crosstalk between subunits can be performed 45, 46, 47. This is not always possible since demetallization occurring during the process of inactivation and other considerations often make the process irreversible. Here, we found for VAP that a relatively high concentration of NaCl throughout the unfolding–refolding cycle was needed and sufficient for reversibility to the active state.

If VAP (and APs in general) utilize a reaction mechanism of the half‐of‐sites type, then the enzyme's activity should be particularly intolerant to changes of residues in interface regions where a network(s) of interactions provide a link between the two active sites. This linking might involve the crystal water molecules observed both in the active sites and at the monomer interface in the crystal structure of VAP (PDB ID: 3E2D) 48 and other APs. Perhaps, a movement or tilting of the dimer interface occurs in each catalytic cycle, similar to the switching between R and T states observed in phosphofructokinase. There, a gap between β‐sheets is filled with water molecules which bridge the long‐range interactions of the main chain C=O and N‐H groups in each monomer by hydrogen bonding 49. In fact, most of the interface mutations made in the present paper resulted in a decrease in catalytic activity, substrate affinity or both. This strengthens arguments for an important coordination of movement across the central area of the interface. This would fit ideas about the presumed asymmetry that must be present at some point in the catalytic cycle and ensure reciprocal coordination. In other words, only one subunit might be active in catalysis while the other subunit has undergone a conformational change in order to release the product from the recently active subunit efficiently. Possibly, such conformational changes in the inactive subunits may be driven by a positive change in entropy through bound water networks that spur on the catalytic events in the active subunit 50.

## Materials and methods

### Materials

Chemicals were obtained from Sigma‐Aldrich (Schnelldorf, Germany) or Merck (Darmstadt, Germany) unless stated otherwise. FluoroPure™ grade mono‐bromobimane (mBrB) was purchased from Molecular Probes (Thermo Fisher, Waltham, MA, USA). Urea ReagentPlus^®^ 99.5% was obtained from Sigma‐Aldrich.

### Generation of enzyme variants

*Vibrio* alkaline phosphatase tagged with StrepTagII (WSHPQFEK) at the C terminus via a two‐amino acid linker (Ser‐Ala) was previously subcloned to a pET11a vector 19. Site‐directed mutagenesis was performed using the QuikChange^®^ method (Stratagene, La Jolla, CA, USA) following the manufacturer's protocol. Oligonucleotide primers were synthesized by TAG (Copenhagen, Denmark). Mutated plasmids were transformed into XL10‐Gold^®^ competent cells (Agilent, Santa Clara, CA, USA) and the plasmids purified on GeneJet^®^ plasmid silica columns (Fermentas, Waltham, MA, USA) using the manufacturer's protocol and then sequenced to confirm the mutated sequences (Genewiz, UK).

### Enzyme production

Enzyme variants were expressed in Lemo21(DE3) cells (New England Biolabs, Ipswich, MA, USA), and purification done in one step on a StrepTactin column by a protocol that has been described elsewhere 19. The enzyme was eluted in 25 mm Tris, pH 8.0, 10 mm MgCl_2_, 2.5 mm desthiobiotin, with 15% v/v ethylene glycol added as a storage medium to prevent frost damage. Enzyme purity was judged to be 95–99% pure by SDS/PAGE on 4–12% bis‐Tris NUPAGE^®^ gels (Life technologies, Carlsbad, CA, USA). Enzymes were snap‐frozen and stored at −20 °C and never used in analysis after more than one freeze/thaw cycle. For denaturation and refolding studies, the enzyme was concentrated on HiTrap Q XL (GE Healthcare, Chicago, IL, USA) ion exchange column on a low‐pressure Bio‐Rad BioLogic LP Chromatography System and eluted with a NaCl gradient from 0.0 to 1.0 m and then further concentrated on a 2.0‐mL Amicon Ultra centrifugation filter with a 30k cut‐off (Millipore, Burlington, MA, USA). Concentrated enzyme samples were aliquoted to 10 μL single use portions and stored at −20 °C.

### Fluorescent labelling of enzyme

Enzymes were labelled while protected from light in 20 mm Mops, 1 mm MgSO_4_, pH 7.5 at 4 °C overnight with mono‐bromobimane (mBrB) at 1.0 mm, added from a 100 mm stock in acetonitrile. The concentration of enzyme varied from 25–50 μm. The enzyme was applied to an Amicon^®^ Ultra 2.0‐mL centrifugation filter with a 30 kDa cut‐off (Millipore) to remove excess probe. The dilution factor during filtration was at least 10 000‐fold and was not deemed sufficient unless the probe absorbance at 380 nm was reduced to zero (<0.000). mBrB is mostly nonfluorescent but is broken down to a fluorescent product by photolysis 51. Thus, to make sure the excess unreacted probe was well below background fluorescence of the enzyme bound probe, the fluorescence of the samples was monitored shortly under constant excitation. If the emission rose with time under excitation, then unreacted probe was still present.

Labelling of the enzyme while still bound on the StrepTactin affinity column at the end of the purification procedure was much less efficient than when done in solution. For in‐solution labelling, the labelling efficiency was between 40% and 100% for all variants

Labelling yield was determined by absorbance spectroscopy, using molar extinction coefficients of ε_380_ = 5000 m ^−1^·cm^−1^ and ε_280_ = 61 310 m ^−1^·cm^−1^ for bimane and VAP monomers, respectively. For enzyme variants, where either Trp or Tyr where introduced or substituted, a value of 5500 m ^−1^·cm^−1^ or 1490 m ^−1^·cm^−1^ was either subtracted or added to the enzyme's molar extinction coefficient (ε_280_) for Trp and Tyr, respectively 52. The probe's absorbance at 280 nm (almost neglectable) was subtracted in the calculations for enzyme concentration.

Labelled enzyme variants were generally used directly for analysis, but in some cases, they were snap‐frozen in liquid nitrogen for later analysis. One freezing cycle was shown not to affect the activity of the enzyme or fluorescence of the bimane probe.

### Standard activity assay

Activity assays were performed at 25 °C in 1.0 m diethanolamine, 1 mm MgCl_2_, pH 9.8 using 5 mm para‐nitrophenyl phosphate (pNPP) as substrate by monitoring the absorbance change at 405 nm on an Evolution 220 spectrophotometer (Thermo Scientific) vs. time and applying ε_405 _= 18 500 m ^−1^·cm^−1^. Generally, 10 μL enzyme sample was pipetted to 990 μL of assay buffer.

### Fluorescence measurements

All steady‐state fluorescence measurements were done on a Horiba FluoroMax4‐P (Kyoto, Japan). Trp fluorescence was monitored at 10 °C by exciting samples at 295 nm and scanning emission from 310 to 400 nm using slit widths of 3 nm and 5 nm for excitation and emission, respectively For bimane‐labelled samples, fluorescence was monitored at 10 °C by excitation at 380 nm and the emission measured from 400 to 600 nm using slit width of 3 nm and 5 nm for excitation and emission respectively. The bimane probe absorbs slightly at 295 nm and gives a negligible fluorescence background in the Trp fluorescence measurements. The wavelength of maximum fluorescence (λ_max_) was calculated by fitting the spectra to a 3rd‐degree polynomial (*y* = *ax* ^3^ + *bx* ^3^ + *cx* + *d*, where *y* is the fluorescence intensity and *x* the wavelength) in the range ± 20 nm from the expected value. To acquire λ_max_, the roots of the first derivative were solved using the quadratic equation for the polynomial.

Time‐resolved measurements were performed on a Horiba FluoroMax4‐P equipped with a FluoroHub time‐correlated single photon counting (TCSPC) system. A NanoLED N370 (370 nm ± 10 nm) from Horiba, which gives a pulse width of <1.2 ns, was used as an excitation source at 1 Mhz. The emission monochromator was set to 470 nm with slit width of 5 nm, and emission decay rate was measured at 10 °C. The instrument prompt was acquired using Ludox colloidal silica at a similar channel stop/start count ratio as for the measured decay, using the same settings, except the emission wavelength was set to 370 nm (source excitation maximum). The decay was fitted to a three‐exponential model using deconvolution analysis: *I*(*t*) = α_*1*_· *e* ^−*t/*τ*1*^ *+* α_*2*_ *· e* ^*−t/*τ*2*^ *+* α_*3*_ *· e* ^*−t/*τ*3*^ where *I* is the emission count, α_*i*_ is the pre‐exponential factor of the *i*’th component, *t* is time, and τ_*i*_ is the lifetime for the *i*’th component. Then, the relative amplitudes were derived by *ƒ*_*i*_ *= *α_*i*_ */∑*α_*I*_ *,* where *ƒ* is the relative amplitude and α_*I*_ the sum of all the pre‐exponential factors. The average lifetime, <τ>, could then be derived using the equation: <τ> *= ƒ*_*1*_τ_*1*_ *+ ƒ*_*2*_τ_*2*_ *+ ƒ*_*3*_τ_*3*_. Deconvolution of the prompt was needed to decrease noise in the first channels due to a ‘rebound’ occurring at the detector level where photons hit the photon multiplier tube (PMT) then bounce to the detector's glass and then hit again the PMT (this is known by the manufacturer to occur with the N370 LED while using PMT of the R928P type (Personal communication).

### Enzyme kinetics

Steady‐state kinetics were conducted at 10 °C in 0.10 m Caps, 0.50 m NaCl, 1 mm MgCl_2_, pH 9.8. Concentration of para‐nitrophenyl phosphate (pNPP) was varied between 0 and 2.0 mm and the initial activity rate measured by the absorbance change at 405 nm over 30 s period using a temperature‐controlled Evolution 220 spectrophotometer (Thermo Scientific). The exact concentration of pNPP was measured a few hours later after full hydrolysis of the substrate in the cuvettes, by measuring the absorbance at 405 nm using ε_405 _= 18 500 m ^−1^·cm^−1^. Enzyme concentration was measured at 280 nm using ε_280 _= 61 310 m ^−1^·cm^−1^ for VAP monomers. *K*_M_ and *k*_cat_ were determined by nonlinear fit to the Michaelis–Menten equation using graphpad prism ^®^ (San Diego, CA, USA).

### Urea denaturation

Samples were incubated overnight (16 h) at 10 °C in 20 mm Mops, 1 mm MgSO_4_, pH 8.0 or 20 mm Caps, 1 mm MgSO_4_, pH 10.5 with urea ranging from 0.0 to 6.0 m (added from 8.0 m stock). For bimane‐labelled variants, the samples were diluted to A_380_ = 0.001, where the dilution factor from labelled stock was at least 20‐fold (stock A_380 _> 0.020). When only Trp fluorescence of the wild‐type was monitored, the enzyme concentration was generally 0.04 mg·mL^−1^, but ranged from 0.02 to 0.08 mg·mL^−1^ for bimane‐labelled samples. Enzyme activity after urea incubation was measured using the standard activity assay. Two‐state curves were fitted with the ‘sigmoidal with variable slope’ function in graphpad prism, and three‐state curves were fitted using the open‐source software cdpal 53. These fits were only meant to aid the eye. Free energy for denaturation was derived using linear extrapolation (see below).

### Refolding experiments

Enzyme samples were diluted to 0.65 mg·mL^−1^ from a 24 mg·mL^−1^ stock to a buffer containing 6 m urea, 20 mm Mops, 1 mm MgSO_4_ at pH 8.0 and incubated overnight (16 h) at 10 °C. For samples measured at pH 10.5, 20 mm Caps buffer was used instead of Mops. Samples were then diluted 17‐fold in the same buffer, ranging from 0.35 to 6.0 m urea. The samples were then assayed for Trp fluorescence and activity using the standard activity assay both described above.

### Analytical size exclusion chromatography

For evaluation of dimer/monomer populations upon refolding, samples were loaded onto Superose 12 column (GE Healthcare) on a Pharmacia FPLC apparatus. A 20 μL enzyme sample (24 mg·mL^−1^) was denatured by diluting the sample with 15 μL of 8.0 m urea and 5 μL of 0.16 m Mops with 8 mm MgSO_4_, pH 8.0 (10 °C) giving final concentrations of 3.0 m urea, 20 mm Mops, 1 mm MgSO_4_. The addition of 0.5 m NaCl was also tested. The sample was incubated at 10 °C for 16 h and then diluted by a factor of 15x in the same buffer without urea, giving final concentration of 0.20 m urea, and incubated for 4 h at 10 °C before injecting 0.5 mL of the sample to the column. The chromatography buffer used was the same as for the diluted sample (0.20 m urea, 20 mm Mops, 1 mm MgSO_4_, pH 8.0). For experiments at pH of 10.5, 20 mm Caps was used as a buffer, but the samples were treated identically as at pH 8.0. Enzyme control samples were loaded at the same conditions without urea denaturation treatment but were equilibrated for 4 h in the chromatography buffer before loading to the column.

### Thermal inactivation/denaturation

Thermal inactivation/denaturation was monitored by either the loss of activity or secondary structure by circular dichroism spectroscopy (CD). CD spectra were recorded on Jasco J‐1100 CD spectrophotometer (Tokyo, Japan) equipped with PTC‐517 peltier‐thermostatted cell holder. CD spectra were obtained from 190 to 250 nm in 10 mm borate, 10 mm MgCl_2_, pH 8.0 and melting temperature measured at 199 nm and 222 nm fixed wavelength in the same buffer in the range of 25–80 °C, with a temperature gradient of 1 °C·min^−1^. Enzyme concentration was 0.3 mg·mL^−1^. High tension (HT) voltage of the photon multiplier tube was not allowed to exceed 600 V.

Thermal inactivation was performed at various buffer conditions (see Fig. 5). Enzyme samples were diluted 1000x fold to a buffer in glass tubes and pre‐equilibrated at 40 °C in a temperature‐regulated water bath, and the enzyme activity was measured using the standard activity assay as a function of time. Data were fitted to either a single exponential decay function or a double exponential decay function using graphpad prism.

### Fitting models for VAP denaturation

Generally, fitting strategies described in Walters *et al*. 54 were used for evaluation of free energy change between enzymatic states. The change in free energy Δ*G*_*i*_ is related to the equilibrium constant *K*_eq_ for the transition (*i*) as such:

(1) $$\Delta G_{i}=-\text{RT}\;\left(K_{\mathrm{eq}}\right)$$

where R is the gas constant and T the absolute temperature.

The linear extrapolation method was used to estimate Δ*G*_*i*_(H_2_O) at zero denaturant concentration for each transition (*i*) 55.

(2) $$\Delta G_{i}=\Delta G_{i}(H_{2O})-m\left[\mathrm{urea}\right]$$

At pH 8.0, VAP unfolding can be described by a three‐state model:

(3) $$N_{2}\overset{K_{1}}{⟶}I_{2}\overset{K_{2}}{⟶}2U$$

where *N*_2_ is the native (fully active) dimer, *I*_2_ a dimer intermediate and *U* the unfolded protein. The equilibrium constant for each transition is described as *K*_*I*_ = [*I*_2_]/[*N*_2_] and *K*_*u*_ = [*U*]^2^/[*I*_2_]. It can be calculated as:

(4) $$K_{I}=\left(1-f_{1}\right)/f_{1}$$

(5) $$K_{u}=2P_{T}{\left(1-f_{2}\right)}^{2}/f_{2}$$

where *P*_*T*_ is the total monomer concentration, ƒ_1_ is the fraction of protein in the native state with ƒ_1_ = [*I*_2_]/[*N*_2_] and ƒ_2_ the fraction in the dimer intermediate state with ƒ_2_ = 2[*I*_2_]/(2[*I*_2_] + [U]). *K*_*I*_ was determined using activity measurements where ƒ_1_ at zero molar urea was set to 1.0 as a relative constant. For *K*_*u,*_ the ƒ_2_ at each measurement was determined using Trp fluorescence by normalizing the signal with the pre/postslope normalization method 55.

For measurements done at pH 10.5, the denaturation was a two‐state process with no populated intermediates:

(6) $$N_{2}\overset{K_{u}}{\to }2U$$

Here, *K*_*u*_ was calculated as in Eqn. (5) but *ƒ*_2_ becomes: *ƒ*_2_ = 2[*N*_2_]/(2[*N*_2_]+[*U*]). Either Trp fluorescence or activity measurements as a function of urea could be used to evaluate *K*_*u*_ at pH 10.5.

## Conflict of interest

The authors declare no conflict of interest.

## Author contributions

JGH and BÁ planned experiments. JGH performed experiments. JGH and BÁ analysed data. BÁ contributed reagents. JGH and BÁ wrote the paper.

## Supporting information


**Table S1.** Inactivation rate constants derived from Figure 5.
**Table S2.** Kinetics of *p*‐nitrophenyl phosphate hydrolysis and efficiency of bimane labelling of VAP variants. Protein concentrations were evaluated using A_280_ by subtracting the label contribution at 280 nm. Bimane concentration was calculated using ɛ_380_ = 5000 m ^‐1^ cm^‐1^. Enzyme kinetics were performed in 0.10 m Caps, 1 mm MgCl_2_, 500 mm NaCl, pH 9.8 °C (10°) C using *p*‐nitrophenyl phosphate (pNPP) as substrate. Data is presented as mean ± SD.
**Table S3.** Bimane time resolved fluorescence measurements. Fluorescence decay was measured using NanoLED N370 source on a Horiba Fluormax 4‐P equipped with a FluorHub TCSPC system and emission monochromator set at 470 nm using 5 nm slit width. Measurements were done in 20 mm Mops, 1 mm MgSO_4_, pH 8.0 at 10 °C. The decay was fitted to a three‐exponential decay model using deconvolution analysis: *I*(*t*) = α_1_· e^−*t*/τ1^ + α_2_·e^−*t*/τ2^ + α_3_· e^−t/τ3^. *f_i_* = ɑ*_i_*/Σɑ*_I_*, ^c^ <τ> = *f*_1_τ_1_ + *f*_2_τ_2_ + *f*_3_τ_3_. χ^2^ is the chi‐squared value of the fit. Unfolded A60Bim/F355W variants was incubated in 4.5 m urea under same conditions before measurement. Note that longer lifetimes for bimane are reported here than observed previously 24, likely due to the emission being recorded at 10 °C compared to 25 °C, where viscosity of water is higher (increased solvent relaxation). Data is presented as mean ± SD.
**Fig. S1.** Bimane fluorescence of the interface residues A60C and K486C in VAP. (A, C) Bimane emission spectra of A60C/F355W, K486C, with bimane attached to the cysteine residues, and their phenylalanine analogs (controls), A60C and K486C/Y42F. (B, D) Lifetime decays of bimane probes at positions K486C and A60C. The instrument prompt is shown in blue. Enzymes were measured in 25 mm Mops, 1 mm MgSO_4_, pH 8.0 at 10 °C.
