# Supplementary material for: Chloride promotes refolding of active Vibrio alkaline phosphatase through an inactive dimeric intermediate with an altered interface
Source: FEBS Open Bio. 2018 Dec 21;9(1):169–84. doi: 10.1002/2211-5463.12565 (PMC6325577; doi:10.1002/2211-5463.12565)
Supplement: Supplementary file 1 — Table S1. Inactivation rate constants derived from Figure 5. Table S2. Kinetics of p‐nitrophenyl phosphate hydrolysis and efficiency of bimane labelling of VAP variants. Protein concentrations were evaluated using A280 by subtracting the label contribution at 280 nm. Bimane concentration was calculated using ɛ380 = 5000 m ‐1 cm‐1. Enzyme kinetics were performed in 0.10 m Caps, 1 mm MgCl2, 500 mm NaCl, pH 9.8 °C (10°) C using p‐nitrophenyl phosphate (pNPP) as substrate. Data is presented as mean ± SD. Table S3. Bimane time resolved fluorescence measurements. Fluorescence decay was measured using NanoLED N370 source on a Horiba Fluormax 4‐P equipped with a FluorHub TCSPC system and emission monochromator set at 470 nm using 5 nm slit width. Measurements were done in 20 mm Mops, 1 mm MgSO4, pH 8.0 at 10 °C. The decay was fitted to a three‐exponential decay model using deconvolution analysis: I(t) = α1· e−t/τ1 + α2·e−t/τ2 + α3· e−t/τ3. fi = ɑi/ΣɑI, c <τ> = f 1τ1 + f 2τ2 + f 3τ3. χ2 is the chi‐squared value of the fit. Unfolded A60Bim/F355W variants was incubated in 4.5 m urea under same conditions before measurement. Note that longer lifetimes for bimane are reported here than observed previously 24, likely due to the emission being recorded at 10 °C compared to 25 °C, where viscosity of water is higher (increased solvent relaxation). Data is presented as mean ± SD. Fig. S1. Bimane fluorescence of the interface residues A60C and K486C in VAP. (A, C) Bimane emission spectra of A60C/F355W, K486C, with bimane attached to the cysteine residues, and their phenylalanine analogs (controls), A60C and K486C/Y42F. (B, D) Lifetime decays of bimane probes at positions K486C and A60C. The instrument prompt is shown in blue. Enzymes were measured in 25 mm Mops, 1 mm MgSO4, pH 8.0 at 10 °C. [file FEB4-9-169-s001.pdf]

Supporting information

Chloride promotes refolding of active Vibrio alkaline phosphatase through an inactive dimeric intermediate with an altered interface

Jens Guðmundur Hjörleifsson and Bjarni Ásgeirsson

Department of Biochemistry, Science Institute, University of Iceland, Dunhagi 3, 107 Reykjavik, Iceland.

Corresponding authors: Jens Guðmundur Hjörleifsson, email: jensgh@hi.is

Table S1: Inactivation rate constants for VAP derived from Figure 5.

	k_1 (min^{-1})	k_2 ($min\text{-}^{1}$)
10 mM Borate	0.18	0.043
10 mM Borate + 1 mM $MgSO_4$	0.10	N/A
10 mM Borate + 10 mM $MgCl_2$	0.13	0.045
10 mM Tris	1.6	0.083
10 mM Tris + 0.1 mM Pi	0.26	0.073
10 mM Tris + 150 mM NaCl	0.16	0.009
10 mM Tris + 10 mM MgCl2	2.0	0.086
10 mM Caps	0.12	N/A

N/A: Not Applicable

Table S2. Kinetics of *p*-nitrophenyl phosphate hydrolysis and efficiency of bimane labelling of VAP variants. Protein concentrations were evaluated using A_{280} by subtracting the label contribution at 280 nm. Bimane concentration was calculated using $\varepsilon_{380} = 5000\ M^{-1}cm^{-1}$. Enzyme kinetics were performed in 0.10 M Caps, 1 mM $MgCl_2$, 500 mM NaCl, pH 9.8 °C (10°) C using *p*-nitrophenyl phosphate (pNPP) as substrate. Data is presented as mean ± SD.

	Labeling efficiency (mol/mol)	λ_{max} abs (nm)	λ_{max} emm (nm)	k_{cat} (s^{-1})	K_M (μM)	k_{cat}/K_M ($s^{-1}M^{-1}$)	k_{cat} (labeled) (s^{-1})	K_M (labeled) (μM)	k_{cat}/K_M (labeled) ($s^{-1}M^{-1}$)
**WT**	-	-	-	302 ± 20	194 ± 30	1.6×10^6	-	-	-
**A60C**	0.63	382	468	105± 7	373 ± 27	2.8×10^5	41 ± 7	213 ± 22	1.9×10^5
**F355W**	-	-	-	255 ± 56	218 ± 20	1.2×10^6	-	-	-
**A60C/F355W**	1.05	387	464	6 ± 2	314 ± 19	1.9×10^4	2 ±1	185 ± 21	1.1×10^4
**K486C**	0.43	381	473	195 ± 12	138 ± 36	1.4×10^6	265 ± 27	250 ± 24	9.7×10^5
**Y42F**	-	-	-	262 ± 33	258 ± 29	1.0×10^6	-	-	-
**K486C/Y42F**	0.60	383	473	83 ± 10	134 ± 9	6.2×10^5	91 ± 7	167 ± 21	5.4×10^5

Table S3. Bimane time resolved fluorescence measurements. Fluorescence decay was measured using NanoLED N370 source on a Horiba Fluormax 4-P equipped with a FluorHub TCSPC system and emission monochromator set at 470 nm using 5 nm slit width. Measurements were done in 20 mM Mops, 1 mM $MgSO_4$, pH 8.0 at 10 °C. The decay was fitted to a three-exponential decay model using deconvolution analysis: $I(t) = \alpha_1 \cdot e^{-t/\tau 1} + \alpha_2 \cdot e^{-t/\tau 2} + \alpha_3 \cdot e^{-t/\tau 3}$. $f_i = \alpha_i/\sum\alpha_I$, [c] $\langle\tau\rangle = f_1\tau_1 + f_2\tau_2 + f_3\tau_3$. χ^2 is the chi-squared value of the fit. Unfolded A60Bim/F355W variants was incubated in 4.5 M urea under same conditions before measurement. Note that longer lifetimes for bimane are reported here than observed previously [24], likely due to the emission being recorded at 10°C compared to 25°C, where viscosity of water is higher (increased solvent relaxation). Data is presented as mean ± SD.

**Enzyme variant**	**τ_1 (ns)[a]**	**τ_2 (ns)**	**τ_3 (ns)[d]**	**f_1[b]**	**f_2**	**f_3**	**<τ> (ns)**	**χ^2**
A60Bim	1.4 ± 0.1	8.0 ± 0.4	19.0 ± 0.2	0.45	0.38	0.17	6.9	1.3
A60Bim/F355W	1.8 ± 0.1	8.1 ± 0.9	19.1± 0.3	0.49	0.36	0.15	6.6	1.2
Unfolded A60Bim/F355W	4.0 ± 1.7	9.7 ± 3.0	13.4 ± 0.6	0.18	0.48	0.35	10.0	1.2
K486Bim	0.8 ± 0.1	6.7 ± 0.4	17.0 ± 0.2	0.46	0.38	0.17	5.8	1.2
K486Bim/Y42F	0.9 ± 0.1	7.2 ± 0.3	16.9 ± 0.2	0.51	0.35	0.13	5.2	1.2

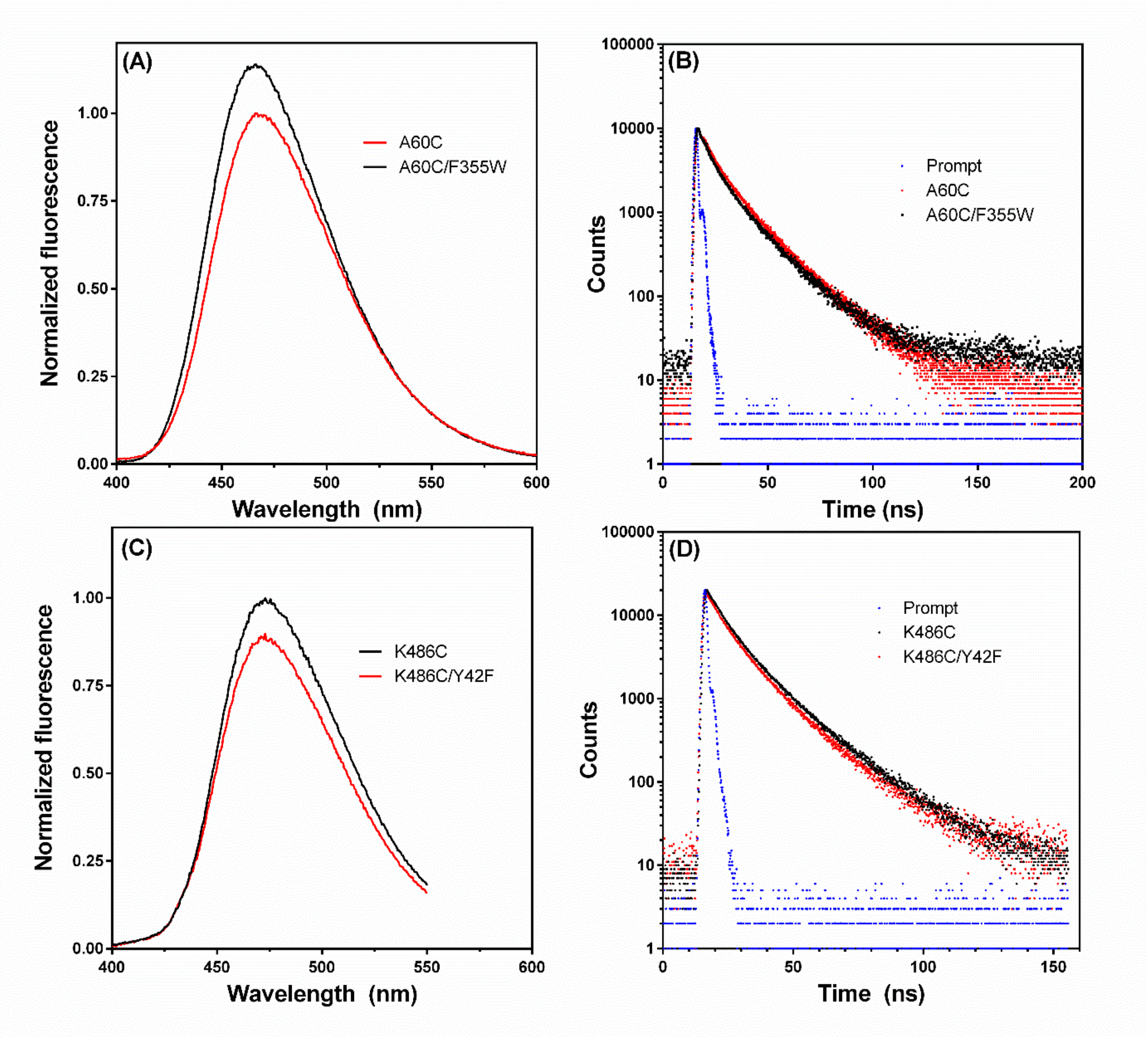

Figure S1. Bimane fluorescence of the interface residues A60C and K486C in VAP. (A, C) Bimane emission spectra of A60C/F355W, K486C, with bimane attached to the cysteine residues, and their phenylalanine analogs (controls), A60C and K486C/Y42F. (B, D) Lifetime decays of bimane probes at positions K486C and A60C. The instrument prompt is shown in blue. Enzymes were measured in 25 mM Mops, 1 mM $MgSO_4$, pH 8.0 at 10°C.
